# FPGA-Parallelized Digital Filtering for Real-Time Linear Envelope Detection of Surface Electromyography Signal on cRIO Embedded System

**DOI:** 10.3390/s25216770

**Published:** 2025-11-05

**Authors:** Abdelouahad Achmamad, Atman Jbari, Nourdin Yaakoubi

**Affiliations:** 1 Laboratoire d’Acoustique de l’Universite du Mans (LAUM), UMR CNRS 6613, Institut d’Acoustique-Graduate School (IA-GS), CNRS, Le Mans Universite, 72085 Le Mans, France; nourdin.yaakoubi@univ-lemans.fr; 2 Electronic Systems Sensors and Nano-Biotechnologies, National Graduate School of Arts and Crafts (ENSAM), Mohammed V University in Rabat, Rabat 8007, Morocco; atman.jbari@ensam.um5.ac.ma

**Keywords:** electromyography, linear envelope, CompactRIO, LabVIEW, FPGA, low pass filter, moving average filter, discrete implementation structure

## Abstract

Surface electromyography (sEMG) signal processing has been the subject of many studies for many years now. These studies had the main objective of providing pertinent information to medical experts to help them make correct interpretations and medical diagnoses. Beyond its clinical relevance, sEMG plays a critical role in human–machine interface systems by monitoring skeletal muscle activity through analysis of the signal’s amplitude envelope. Achieving accurate envelope detection, however, demands a robust and efficient signal processing pipeline. This paper presents the implementation of an optimized processing framework for the real-time linear envelope detection of sEMG signals. The proposed pipeline comprises three main stages, namely data acquisition, full-wave rectification, and low-pass filtering, where the deterministic execution time of the algorithm on the FPGA (98 ns per sample) is two orders of magnitude faster than the data acquisition sample interval (200 µs), guaranteeing real-time performance. The entire algorithm is designed for deployment on the FPGA core of a CompactRIO embedded controller, with emphasis on achieving high accuracy while minimizing hardware resource consumption. For this purpose, a parallel second-order structure of the Butterworth low-pass (LP) filter is proposed. The designed filter is tested and compared practically to the conventional method, which is the moving average (MAV) filter. The mean square error (MSE) is used as a metric for performance evaluation. From the analysis, it is observed that the proposed design LP filter shows an improved MSE and reduced hardware resources than the MAV filter. Furthermore, the comparative analysis and the results show that our proposed design LP filter is a valid and reliable method for linear envelope detection.

## 1. Introduction

In recent years, significant efforts have been devoted to electromyography (EMG) signal processing to meet the evolving demands of the kinesiology and biomechanics fields [1]. As the growing demand for intelligent and responsive technological solutions continues to rise, the need for integrated EMG systems designed with precision and efficiency has become increasingly vital, particularly within the healthcare system [2]. In this regard, a thorough understanding of the underlying physiology and neuromuscular system serves as an original way to introduce the concept of the EMG signal processing and acquisition. Muscle force is mainly controlled through the central nervous system (CNS) [3]. While the human body contains various muscle types, skeletal muscle is the primary focus of extensive research, especially for applications like muscle fatigue assessment [4] and rehabilitation [5]. Skeletal muscle comprises numerous muscle fibers (MFs), each innervated by motor neurons. An individual alpha motor neuron sends its axon to innervate multiple MFs, activating them synchronously and directly triggering their contraction [3]. In fact, sEMG itself represents the collective electrical potentials generated by skeletal muscle during contraction, which represent neuromuscular activity [6]. This bio-signal results from the accumulation of motor unit action potential (MUAP) trains detected near recording electrodes [3,7]. The electrical activity of muscle can be measured with electrodes that are either placed on the skin surface of the muscle of interest, known as surface electromyography (sEMG), or inserted into the muscle, known as intramuscular electromyography (iEMG) [6]. Among the available methods to evaluate skeletal muscle, sEMG is more appropriate because it is easy to apply and it does not cause any suffering to patients [1]. For the sEMG signal, its power spectrum is mostly concentrated in the range between 10 Hz to 250 Hz and the amplitude is a few millivolts [3]. Although information-rich, the raw sEMG signal often provides more detail than typically needed for human–machine interface (HMI) applications, which focus primarily on muscle activation status and contraction intensity. To this aim, the sEMG signal envelope is the standard choice in such contexts rather than the raw signal [8].

Therefore, a crucial piece of information derived from the sEMG signal is its envelope. The extraction of the linear envelope during contraction and relaxation movements is a fundamental first step for applications such as prosthesis control and robotic systems, as it facilitates the evaluation of muscle activity amplitude [9]. Additionally, the linear envelope also serves an important function in detecting the onset and offset of muscle activity [8,10].

In the field of clinical diagnosis, estimating the onset and the offset of the sEMG signal is useful to diagnose a certain abnormality in muscle coordination [11] and to detect some pathological, noisy, and weak myoelectric signals [10]. Nevertheless, finding an accurate and robust linear envelope appears to be paradoxical and experimental. A visual inspection of the sEMG envelope needs high dexterity and clinical experience. Over the last decades, many technical methods have been reported in the literature to address the linear envelope enhancement employing the moving average (MAV) [12,13], Hilbert transform (HT) [14], root mean square (RMS) [11], low-pass (LP) filter [8,11,13], and discrete wavelet transform (DWT) [15], among many others. While advanced adaptive methods exist for optimizing envelope extraction [16,17], many current methods are not suitable for real-time operation, mainly due to their heavy computational burden. This raises significant limitations for wearable and portable HMI applications, which rely on battery-powered devices. Such devices typically use resource-constrained computing platforms to achieve very low power consumption and are therefore unable to support computationally intensive tasks. However, there is growing interest in the development of real-time processing algorithms specifically optimized for embedded systems. These systems, based on programmable hardware (e.g., FPGAs, SoCs), operate within the paradigm of edge computing.

**Figure 1:**
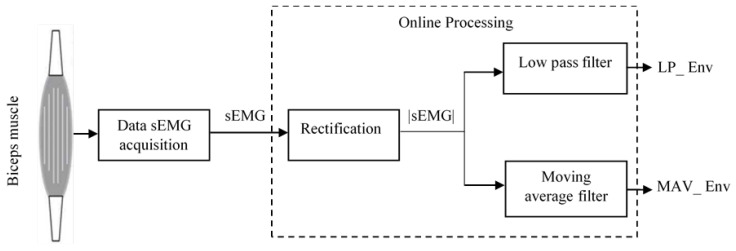
Standard diagram of linear envelope detection.

In the present paper, a simple and elegant solution for the linear envelope detection of sEMG signals is implemented through a cRIO-FPGA chip, with extremely low computational complexity. Figure 1 illustrates the linear envelope detection process. The method comprises the three following sequential steps: (1) initial pre-processing of the raw sEMG signal within the acquisition system, (2) full-wave rectification via absolute value conversion, and (3) filtering of the rectified signal using either a custom-designed low-pass (LP) filter or a moving average value (MAV) filter.

The objective of our study is to examine and compare, from a practical point of view, our proposed design for an LP filter and a conventional MAV filter in terms of linear envelope detection accuracy and the quantity of hardware resources used. Although the LP filter is well-known in the literature, its use for linear envelope detection has never been investigated thoroughly. It is likely that this is due to the fact that the frequency response of the LP filter causes significant distortion to the sEMG spectrum. Nevertheless, this study shows that the parallel LP filter is effective for linear envelope detection. Given that this level of performance is attained with the computational cost of a single sum per sample, the LP filter presents substantial computational advantages over alternative filtering approaches in HMI applications. Consequently, it is readily implementable on highly resource-constrained, low-power platforms and can free computational resources for more intensive tasks. To the best of our knowledge, implementing a parallel LP filter on a cRIO-FPGA chip has never been investigated in the EMG signal processing domain. Thus, the contributions of our study can be summarized as follows:

- Determining the best design of an efficient infinite impulse response (IIR) digital filter for linear envelope detection. To do so, the filter designing and analysis tool (FDATool) is used. This powerful MATLAB R2023a interface provides a detailed description and analysis that address the requirement of a correctly designed digital filter.
- Converting the standard direct form I IIR filter into a parallel structure composed of second-order sections via partial fraction expansion (PFE).
- Proposing a novel practical methodology for comparative performance assessment of the filtering techniques.

This paper is organized as follows: Section 2 presents a theoretical background of the proposed design of the LP filter. Section 3 presents the proposed experimental layout and the selected NI hardware/software (LabVIEW 2023 Q1) implementation. Section 4 describes the experimental results and the comparative analysis. Section 5 is reserved for the conclusion.

## 2. Design and Parallel Implementation of Digital Filter

### 2.1. Basic Structure of Low-Pass Filter

After the sEMG signal is rectified, a digital low-pass filter is applied to extract its envelope. Among the various filter types, the infinite impulse response (IIR) filter stands out as an effective option in digital signal processing (DSP). Also known as a recursive filter, the IIR filter offers significant advantages, including a reduced number of coefficients, lower computational complexity, and minimal memory requirements. However, these benefits come at the cost of a non-linear phase response. Despite this limitation, IIR filters are widely used in numerous signal processing applications such as automatic control, communication systems, speech processing, and pattern recognition [18].

(1) $$y\left(i\right)+\sum_{k=1}^{N}a_{k}y\left(i-k\right)=\sum_{k=0}^{M}b_{k}x\left(i-k\right)$$

By applying the Z-transform to the difference equation and using the shifting property of the Z-transform, we derive the corresponding transfer function in the Z-domain:

(2) $$H\left(z\right)=\frac{Y(z)}{X(z)}=\frac{\sum_{k=0}^{M}b_{k}z^{-k}}{1+\sum_{k=1}^{N}a_{k}z^{-k}}$$

where X(z) denotes the Z-transform of the input signal, while Y(z) is the Z-transform of the output signal (filtered signal), $a_{k}$ and $b_{k}$ are the filter coefficients for the recursive and feedforward parts, respectively, and $a_{0}=1$ is a standard convention for normalizing the transfer function. The parameter *M* is the number of zeros, and *N* is the number of poles. To ensure the transfer function is proper (i.e., the degree of the numerator does not exceed that of the denominator), it is required that N≥M.

### 2.2. Design and Specifications

**Figure 2:**
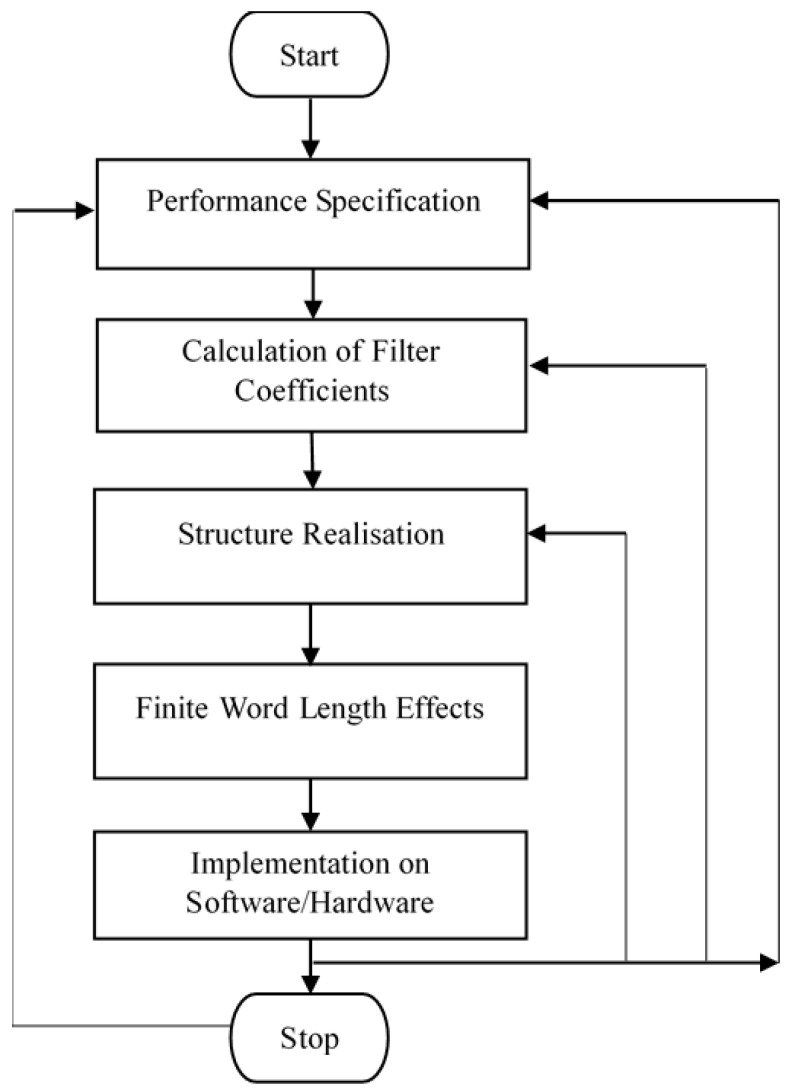
Flowchart of digital filter design.

One significant challenge in LP filter design lies in accurately determining the optimal filter coefficients for linear envelope detection. The digital IIR filter design process, illustrated in Figure 2, consists of five distinct stages [19].

Most related studies emphasize the importance of carefully selecting the cut-off frequency, which should typically be set below 10 Hz to ensure optimal performance [20]. Such specifications for the LP filter are crucial for producing a smooth and accurate linear envelope of the surface sEMG signal. Guided by these recommendations and supported by extensive trial-and-error experimentation, the final parameters and corresponding values of the proposed filter design are summarized in Table 1.

Using the selected parameters for the IIR filter, the optimal coefficients $a_{k}$ and $b_{k}$ of the transfer function H(z) were computed using MATLAB’s FDATool interface within the Signal Processing Toolbox in MATLAB R2023a. The resulting coefficients are presented in Table 2. Subsequently, the filter analysis stage involved verifying the critical performance characteristics of the designed filter. This included a stability check, confirmed by ensuring all poles of the transfer function lay within the unit circle, and an analysis of the frequency response to ensure it met the specified cut-off and attenuation requirements.

### 2.3. Parallel Structure of LP Filter

In the DSP domain, IIR filters can be implemented in various structures (e.g., direct, cascade, parallel, lattice). The parallel second-order section structure offers benefits including low quantization noise and high speed on multicore processors, as well as efficiency for parallel implementation on graphics processing units (GPUs) [21,22]. In many applications, converting series biquad filters into a delayed parallel form is advantageous. This conversion is typically performed using partial fraction expansion (PFE), which decomposes the filter’s transfer function into a sum of parallel second-order sections. However, PFE is generally effective only for filters of relatively low order [21]. Therefore, to determine such a decomposition amounts to breaking down the transfer function H(z) of the Equation (2) into a sum of rational fractions. The general system transfer function is

(3) $$H\left(z\right)=\frac{Y(z)}{X(z)}=\sum_{i=1}^{n}H_{i}\left(z\right)$$

**Figure 3:**
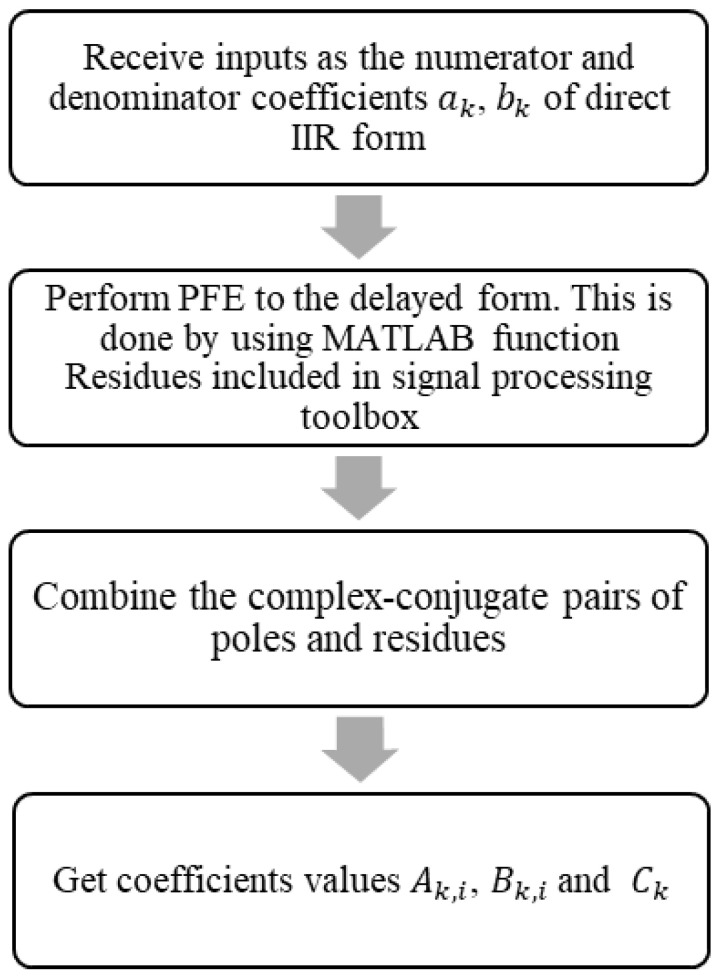
Flowchart for parallel-form coefficient calculation.

Each component $H_{i}\left(z\right)$ is typically a first-order or second-order rational function, derived from PFE. The main recursive part of the transfer function is defined as

(4) $$H_{1}=\sum_{k=1}^{L}\frac{B_{k,0}+B_{k,1}z^{-1}}{1+A_{k,1}z^{-1}+A_{k,2}z^{-2}},k=1,\ldots ,L.$$

where $L=\left\lceil \frac{N}{2}\right\rceil$ denotes the number of second-order sections. It ensures that for an even-order filter (*N* even), $L=\frac{N}{2}$, and for an odd-order filter (*N* odd), $L=\frac{N+1}{2}$, with one section being first-order. $A_{k,1}$, $A_{k,2}$$B_{k,0}$, and $B_{k,1}$ are real-valued coefficients associated with each section. An additional non-recursive component must be included only if M≥N:

(5) $$H_{2}=\sum_{k=0}^{M-N}C_{k}z^{-k}$$

where $C_{k}$ represents the coefficients of the direct-form components. To compute the coefficients $A_{k,i}$, $B_{k,i}$, and $C_{k}$ for the parallel-form decomposition, one must follow the procedural steps illustrated in Figure 3. The figure presents a four-step procedure for converting an IIR filter into its parallel form. It begins by receiving the numerator ($b_{k}$) and denominator ($a_{k}$) coefficients of the filter in direct form. The next step involves applying PFE to the delayed version of the transfer function using MATLAB’s ‘residuez’ function. Complex conjugate pairs of poles and residues are then combined to form real-valued second-order sections. This is achieved by pairing a residue $R_{k}$ and its corresponding pole $P_{k}$ with their complex conjugates, $R_{k}^{*}$ and $P_{k}^{*}$. Finally, the process yields the coefficients $A_{k,i}$, $B_{k,i}$, and $C_{k}$, which represent the recursive and non-recursive parts of the system in parallel form, suitable for hardware implementation. The stability of each resulting second-order section, and therefore the overall parallel filter, was validated by ensuring that all poles lie within the unit circle in the Z-plane.

The obtained coefficients are

$$A_{k,i}=\left[\begin{array}{cc} 1 & 1\\ -1.93529 & -0.93906\\ 0.939120 & 0 \end{array}\right]$$

$$B_{k,i}=\left[\begin{array}{cc} -0.06285 & 0.06291\\ 0.06272 & 0 \end{array}\right]$$

$$C_{k}=\left[\begin{array}{c} -3.3050\times 10^{-5} \end{array}\right]$$

where the first row of $A_{k,i}$ and $B_{k,i}$ corresponds to the second-order section, and the second row corresponds to the first-order section. Based on these coefficients, the transfer function in the parallel second-order form can be written as

(6) $$H\left(z\right)=-3.3050\times 10^{-5}+\frac{0.06291}{1-0.93906z^{-1}}+\frac{-0.06285+0.06273z^{-1}}{1-1.93529z^{-1}+0.9391z^{-2}}$$

**Figure 4:**
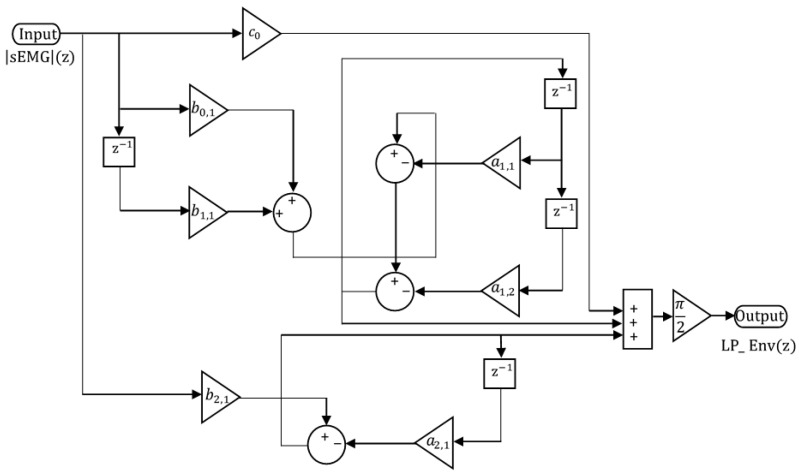
Parallel structure of the LP filter.

Finally, the corresponding Simulink model that implements this parallel form is illustrated in Figure 4.

## 3. Experimental System

### 3.1. The Architecture of an Experimental System

**Figure 5:**
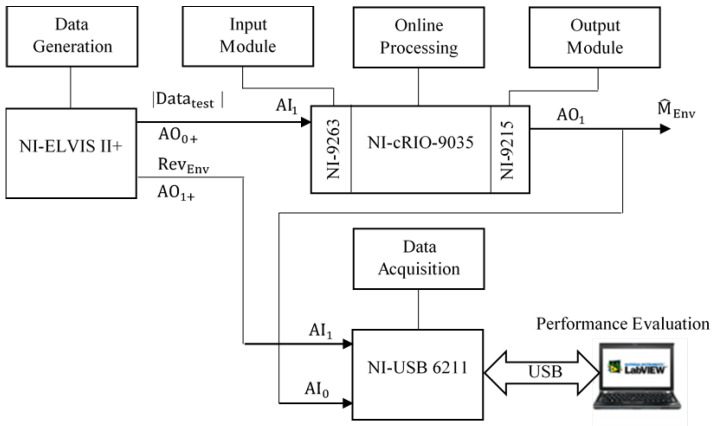
Block diagram for comparison and performance evaluation.

The block diagram illustrated in Figure 5 demonstrates a rigorous comparison between the proposed LP filter design and the MAV filter method. The mean square error (MSE) is used as the primary metric to determine the most suitable filtering technique for the sEMG linear envelope. In this subsection, we briefly introduce the system’s functionality and describe its implementation using National Instruments (NI) hardware and software.

The system operates as follows: first, test data is generated by the NI ELVIS II+ platform and is transmitted to the NI 9263 data acquisition input module. After data acquisition, the signal is processed by the embedded system NI CompactRIO-9035 (NI, Austin, TX, USA). The processed linear envelope is output through the NI 9215 module, where it is compared with a reference envelope. The reference envelope is generated using the NI ELVIS II+ board once again. Finally, the data acquisition NI USB 6211 device transmits the results to a computer via USB for performance evaluation.

**Figure 6:**
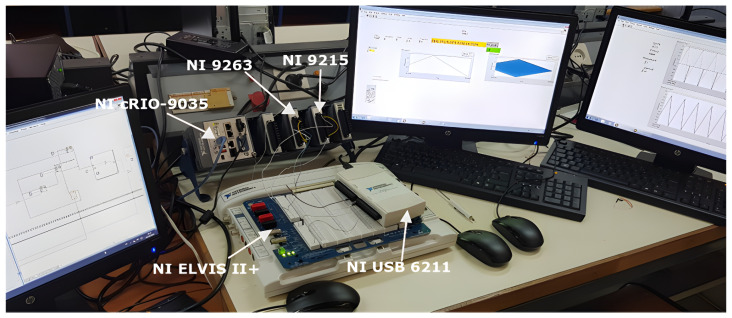
Developed test bench setup, highlighting the key hardware components: the CompactRIO-9035 controller (NI, Austin, TX, USA), the NI 9263 analog output module, and the NI 9215 analog input module.

The selection of equipment is crucial for effective sEMG data acquisition and processing. Figure 6 illustrates the NI hardware instrumentation used in this test bench setup, with particular emphasis on the CompactRIO-9035 (NI, Austin, TX, USA). This device is a cost-effective, reconfigurable control and acquisition system designed to deliver high performance and reliability. It features an open embedded architecture that combines compact size with exceptional robustness for real-time data processing.

As shown in the figure, the CompactRIO-9035 (NI, Austin, TX, USA) integrates a field-programmable gate array (FPGA) triggered by a 40 MHz clock, a real-time processor, and reconfigurable I/O (RIO) technology within a single chassis. The NI 9263 and NI 9215 modules utilized in this study serve as simultaneous analog output and input modules, respectively. Both modules can be easily inserted into the CompactRIO-9035 (NI, Austin, TX, USA) chassis or any other CompactRIO chassis. Additionally, each module provides four channels with 16-bit resolution and supports sampling rates up to 100 kS/s. Each channel is compatible with 5 V/TTL signals.

### 3.2. Digital Filter Implementation on CompactRIO FPGA

A general FPGA architecture comprises configurable I/O blocks, configurable logic blocks (CLBs), and a programmable interconnection network. One of the key advantages of FPGAs is their abundant resources for implementing real-time DSP algorithms. Additionally, the LabVIEW FPGA module provides significant advantages for designing FPGA-based DSP systems.

**Figure 7:**
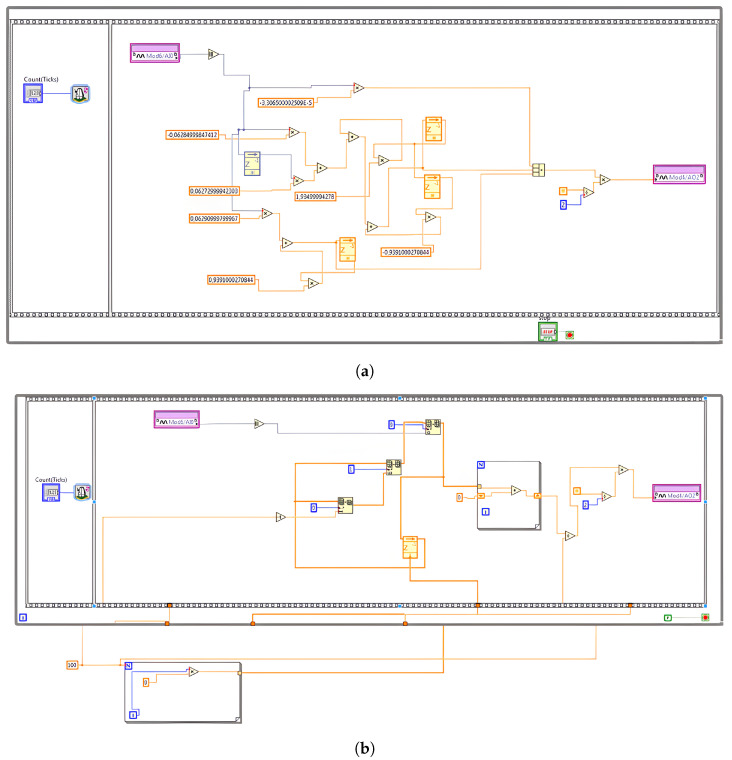
Acquisition and online processing on NI-cRIO FPGA chip: (**a**) proposed design for LP filter; (**b**) MAV filter.

In this study, the virtual instrument (VI) for the FPGA is developed using LabVIEW FPGA targeting the Xilinx Kintex-7 7K70T (AMD, Santa Clara, CA, USA) device. Beyond data acquisition, the FPGA also performs online processing tasks such as full-wave rectification and filtering. As illustrated in Figure 7, the block diagram features a loop containing two frames within a sequence structure. The first frame includes a loop timer that sets the sampling delay to 8000 ticks, while the second frame handles the FPGA input/output nodes. Here, the ticks correspond to pulses on the FPGA clock and can be calculated as follows:

(7) $$Count_{ticks}=\frac{FPGA_{clock}}{F_{s}}=\frac{40\times 10^{6}}{5000}=8000ticks$$

## 4. Results and Discussion

### 4.1. The Proposed Criterion for Performance Evaluation

In this section, we present the results of the implementation and experimental validation of the linear envelope pipeline. The accuracy of the linear envelope detection methods is evaluated using statistical metrics. Specifically, the mean square error (MSE) is employed as the performance criterion for each filtering method. The MSE represents the average of the squared differences between the actual and measured values. Assuming $Ref_{Env}$ is the reference envelope of the test data and ${\hat{M}}_{Env}$ is the corresponding measured envelope, the MSE over *n* samples is defined by Equation (8).

(8) $$MSE=\frac{1}{n}\sum_{i=1}^{n}{\left(Ref_{Env}\left(i\right)-{\hat{M}}_{Env}\left(i\right)\right)}^{2}$$

### 4.2. Reference Envelope Synthesis and Filter Benchmarking

A performance evaluation of sEMG linear envelope detection filters requires a known benchmark. Since no standardized reference exists, we propose a synthetic approach using amplitude modulation (AM) principles. The methodology establishes the following:

- Reference Envelope ($Ref_{Env}\left(t\right)$):
  (9) $$Ref_{Env}\left(t\right)=\left|A_{c}\cdot \mathrm{tri}\left(2\pi f_{m}t\right)\right|$$
  where tri(·) is a triangle wave-modulating waveform, $A_{c}$ is its amplitude, and $f_{m}$ is its frequency. The absolute value ensures a physically valid, non-negative envelope.
- Test Data ($Data_{test}\left(t\right)$):
  (10) $$Data_{test}\left(t\right)=A_{m}\cdot Ref_{Env}\left(t\right)\cdot \sin\left(2\pi f_{c}t\right)$$
  generated by amplitude-modulating a carrier wave ($\sin(2\pi f_{c}t)$) with $Ref_{Env}\left(t\right)$, scaled by amplitude $A_{m}$.

**Figure 8:**
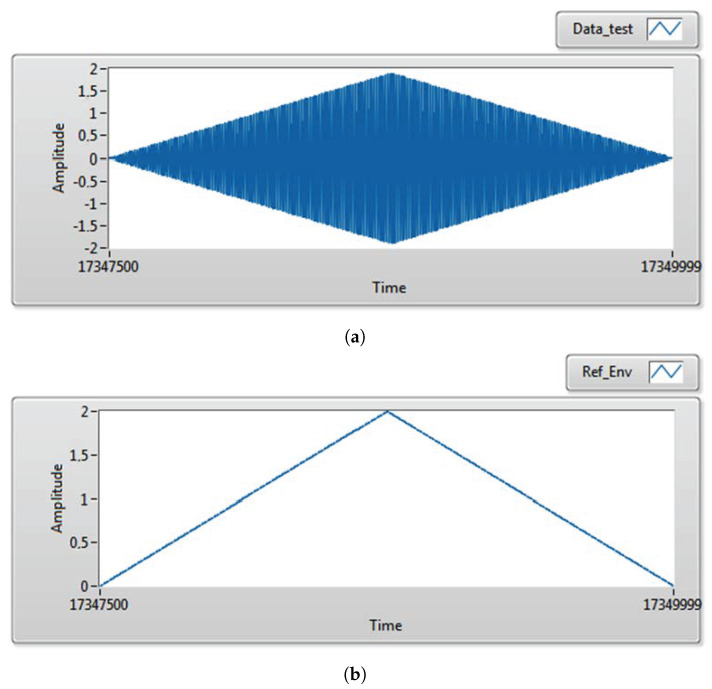
Generated data for evaluation: (**a**) data test; (**b**) reference envelope.

Both signals are synthesized programmatically in LabVIEW 2023 Q1. Figure 8 shows representative $Data_{test}\left(t\right)$ and $Ref_{Env}\left(t\right)$ waveforms.

**Figure 9:**
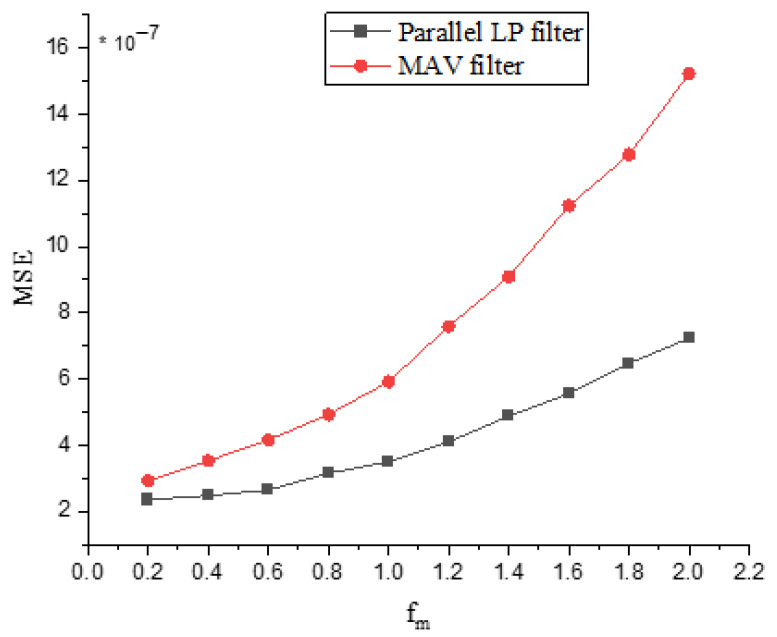
MSE versus frequency of reference envelope.

Figure 9 demonstrates the significant impact of reference envelope frequency on MSE for both the MAV filter and the proposed LP filter, showing that the MSE increases approximately linearly with frequency. This linear relationship indicates a fundamental sensitivity of envelope detection accuracy to higher frequencies. The MAV filter exhibits pronounced sensitivity, meaning its performance degrades severely as frequency rises. In contrast, the proposed LP filter demonstrates significantly better robustness: it maintains consistently lower MSE values than the MAV filter across all frequencies, and its performance degrades much more gradually with increasing frequency. Consequently, while higher reference envelope frequencies universally degrade linear envelope detection accuracy (increased MSE), the proposed LP filter offers a clear performance advantage over the MAV filter, particularly at higher operating frequencies.

### 4.3. Generation and Evaluation of Real sEMG Signal

**Figure 10:**
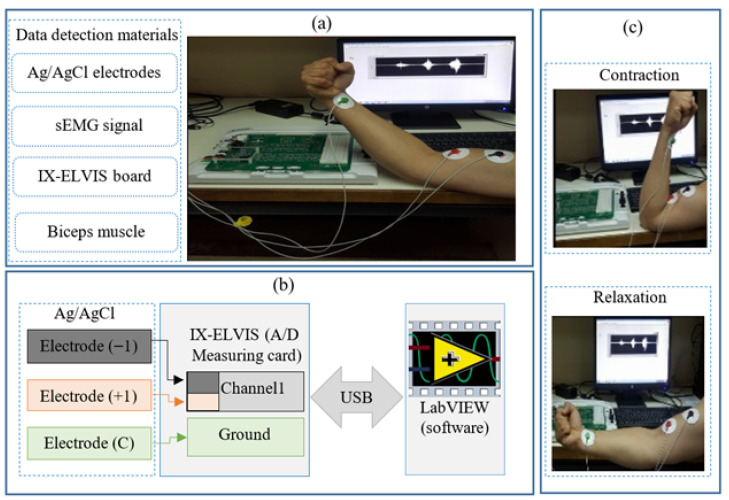
Experimental setup: (**a**) surface electrode placement on bicep brachii muscle following SENIAM guidelines; (**b**) hardware description; (**c**) process of skeletal muscle contraction and relaxation.

**Figure 11:**
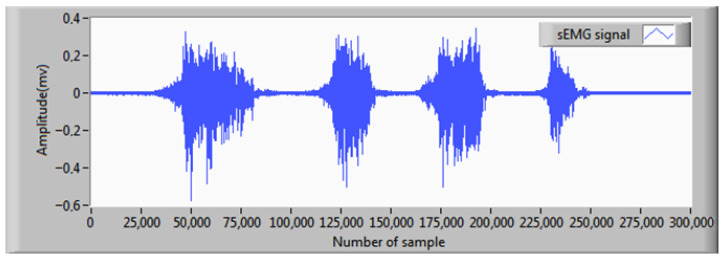
Recorded surface sEMG signal.

As depicted in Figure 10, the sEMG signal is recorded from the bicep brachii muscle of a healthy male subject performing controlled cycles of isometric contraction and relaxation. sEMG signal detection strictly followed SENIAM (Surface EMG for Non-Invasive Assessment of Muscles) guidelines, including standardized protocols for electrode placement (over the muscle belly, parallel to fiber orientation), inter-electrode distance (typically 20 mm center-to-center), and skin preparation (abrasion and alcohol cleaning to achieve impedance < 10 kΩ) [23]. The acquired raw sEMG signal (Figure 11) was obtained using two pairs of bipolar Ag/AgCl circular electrodes (conductor diameter: typically 5–10 mm, gel contact) connected to a dedicated instrumentation amplifier (IX-ELVIS, (NI, Austin, TX, USA) platform). This amplifier provided high input impedance (>1 GΩ), programmable gain (typically 500–2000 V/V), and common-mode rejection (>100 dB) to minimize noise. The conditioned analog signal was sampled at 5 kHz (exceeding the Nyquist rate for sEMG bandwidth, 20–500 Hz) and digitized at 16 bit resolution (yielding a dynamic range of 96.3 dB) using an integrated A/D converter.

**Figure 12:**
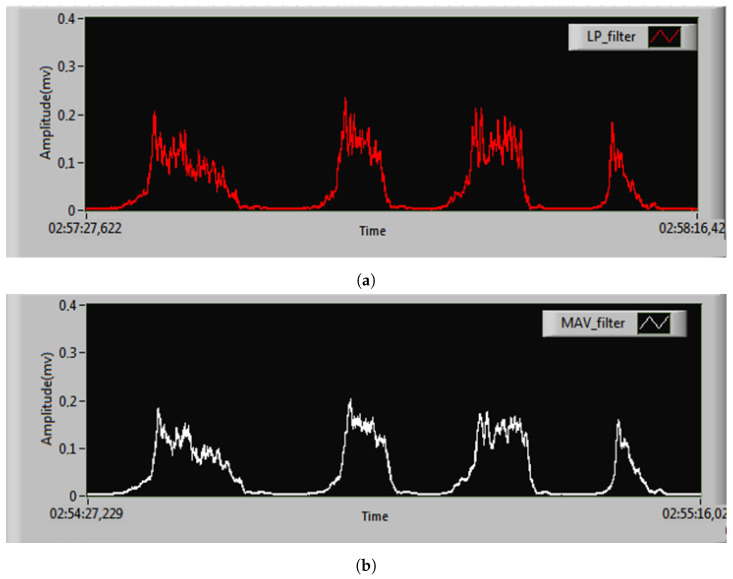
Calculated linear envelope: (**a**) proposed design for LP filter; (**b**) MAV filter with $N_{w}$ = 100 ms.

The real-time linear envelopes from the moving average value (MAV) filter and the proposed LP filter design are compared in Figure 12. The selection of the MAV window length $N_{w}$ critically determines the smoothing level of the linear envelope. Excessively large $N_{w}$ values cause aggressive smoothing that risks attenuating physiologically relevant, rapid muscle activations essential for accurate envelope representation. Conversely, overly small $N_{w}$ values provide insufficient attenuation, failing to adequately suppress high-frequency noise components inherent in the raw sEMG signal and potentially distorting the envelope. This parameter $N_{w}$ directly defines the MAV filter’s effective cut-off frequency ($f_{c}$), establishing the boundary between preserved signal components and attenuated noise. Figure 12b illustrates the MAV filter’s performance specifically with a window length of $N_{w}=100$ ms. This value was chosen to equate the MAV filter’s approximate cut-off frequency, $f_{c}≃\frac{1}{2N_{w}\Delta t}$, where the sampling period is Δt = 0.2 ms. Substituting the values yields $f_{c}$ = 5 Hz, directly matching the LP filter’s cut-off.

**Figure 13:**
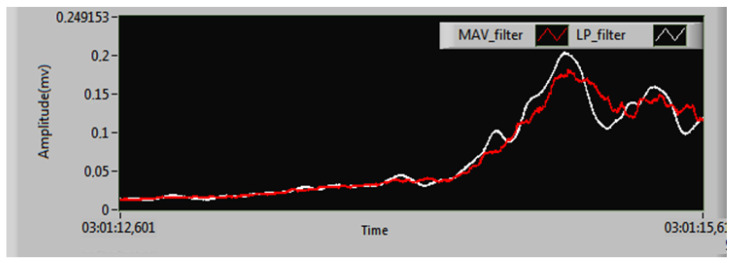
The zoomed linear envelope of our proposed design LP and MAV filter.

Both filters effectively smooth the linear envelope of the sEMG signal (Figure 13), but a visual assessment of accuracy remains challenging. A hardware resource comparison (Table 3) reveals that the proposed parallel LP filter is significantly more efficient than the MAV filter. Implemented on a Xilinx Kintex-7 FPGA, the proposed design uses 2900 slices (28.3% utilization) versus 3894 slices (38%) for the MAV filter—a 25.5% reduction in total slice usage. It also requires 54.7% fewer flip-flops (6635 vs. 14,622) and 22.1% fewer six-input LUTs (7949 vs. 10,207). Block RAM usage is identical (three units, 2.2% utilization), while the proposed design uses six more DSP slices (10 vs. 4). This demonstrates superior overall hardware efficiency for the proposed LP filter despite its higher DSP slice requirement. This increase is attributed to the multiplier-intensive nature of the IIR filter’s parallel structure, which requires several constant-coefficient multiplications per second-order section. In contrast, the MAV filter can be implemented primarily with adders and registers, requiring only a final scaling multiplication (or even a simple bit-shift if the window length is a power of two), thus consuming far fewer DSP slices.

## 5. Conclusions

This study presents a hardware-efficient implementation of linear envelope detection for surface EMG signals, specifically minimizing computational resources while ensuring high accuracy. We practically validated two filtering approaches, the established MAV filter and our custom-designed, optimized LP filter using a NI hardware platform and LabVIEW. Our methodological contribution provides a clear framework for implementing and objectively comparing envelope detection performance. Experimental results demonstrate that our bespoke LP filter achieves superior tracking fidelity while offering significant resource efficiency, utilizing 28.3% of FPGA slices compared to 38% for the MAV filter; a 25.5% reduction in total slice usage. It achieves superior tracking of the underlying muscle activation dynamics with reduced temporal smearing and enhanced signal detail. This precision makes the LP filter particularly advantageous for demanding applications like prosthetic control feedback loops, fine motor rehabilitation assessment, and biomedical research requiring nuanced sEMG analysis.

Future studies will refine the LP algorithm for ultra-low-power embedded deployment and integrate it into comprehensive sEMG processing pipelines. This includes potential synergy with feature extraction, classification stages, and embedded deployment in wearable medical devices.

## Figures and Tables

**Table 1:** IIR filter specifications.

Parameter	Value
Filter type	Low pass
Design method	Butterworth
Structure	Direct form I
Sampling frequency	$F_{s}$ = 5 kHz
Cut-off frequency	$F_{c}$ = 5 Hz

**Table 2:** Generated coefficients for the design of the IIR filter.

*k*	$b_{k}$	$a_{k}$
0	$2.914649446569765\times 10^{-5}$	1
1	$8.743948339709296\times 10^{-5}$	−2.874356892677485
2	$8.743948339709296\times 10^{-5}$	2.756483195225695
3	$2.914649446569765\times 10^{-5}$	−0.881893130592485

**Table 3:** Comparison of hardware resources for the two filtering methods.

FPGA Type	Xilinx Kintex-7 7K70T				
Implemented Filter	Proposed Design LP Filter		MAV Filter		
Device Utilization	Used	%	Used	%	Available
Total slices	2900	28.3	3894	38	10,250
Number of flip-flops	6635	8.1	14,622	17.8	82,000
Number of six-input LUTs	7949	19.4	10,207	24.9	41,000
Block RAMs	3	2.2	3	2.2	135
Number of DSP slices	10	4.2	4	1.7	240

## Data Availability

The original contributions presented in this study are included in the article. Further inquiries can be directed to the corresponding author.

## Abbreviations

The following abbreviations are used in this manuscript:

sEMG	Surface Electromyography
MAV	Moving verage
MSE	Mean Square Error
LP	Low Pass
iEMG	Intramuscular Electromyography
MFs	Muscle Fibers
MUAP	Motor Unit Action Potential
HMI	Human–Machine Interface
RMS	Root Mean Square
DWT	Discrete Wavelet Transform
IIR	Infinite Impulse Response
PFE	Partial Fraction Expansion
NIs	National Instruments
FDATool	Filter Designing and Analysis Tool
DSP	Digital Signal Processing
GPUs	Graphics Processing Units
FPGA	Field-Programmable Gate Array
I/O	Input/Output
CLBs	Configurable Logic Blocks
VI	Virtual Instrument
AM	Amplitude Modulation
SENIAM	Surface EMG for Non-Invasive Assessment of Muscles
